# HIV policy and implementation: a national policy review and an implementation case study of a rural area of northern Malawi^†^

**DOI:** 10.1080/09540121.2016.1168913

**Published:** 2016-04-21

**Authors:** Aisha N. Z. Dasgupta, Alison Wringe, Amelia C. Crampin, Christina Chisambo, Olivier Koole, Simon Makombe, Charles Sungani, Jim Todd, Kathryn Church

**Affiliations:** ^a^ London School of Hygiene and Tropical Medicine, London, UK; ^b^ Karonga Prevention Study, Chilumba,Malawi; ^c^ HIV/AIDS Unit, Ministry of Health, Lilongwe, Malawi; ^d^ Karonga District Health Office, Karonga, Malawi

**Keywords:** Malawi, HIV, policy, implementation, ART

## Abstract

Malawi is a global leader in the design and implementation of progressive HIV policies. However, there continues to be substantial attrition of people living with HIV across the “cascade” of HIV services from diagnosis to treatment, and program outcomes could improve further. Ability to successfully implement national HIV policy, especially in rural areas, may have an impact on consistency of service uptake. We reviewed Malawian policies and guidelines published between 2003 and 2013 relating to accessibility of adult HIV testing, prevention of mother-to-child transmission and HIV care and treatment services using a policy extraction tool, with gaps completed through key informant interviews. A health facility survey was conducted in six facilities serving the population of a demographic surveillance site in rural northern Malawi to investigate service-level policy implementation. Survey data were analyzed using descriptive statistics. Policy implementation was assessed by comparing policy content and facility practice using pre-defined indicators covering service access: quality of care, service coordination and patient tracking, patient support, and medical management. ART was rolled out in Malawi in 2004 and became available in the study area in 2005. In most areas, practices in the surveyed health facilities complied with or exceeded national policy, including those designed to promote rapid initiation onto treatment, such as free services and task-shifting for treatment initiation. However, policy and/or practice were/was lacking in certain areas, in particular those strategies to promote retention in HIV care (e.g., adherence monitoring and home-based care). In some instances, though, facilities implemented alternative progressive practices aimed at improving quality of care and encouraging adherence. While Malawi has formulated a range of progressive policies aiming to promote rapid initiation onto ART, increased investment in policy implementation strategies and quality service delivery, in particular to promote long-term retention on treatment may improve outcomes further.

## Background

The scale-up of antiretroviral therapy (ART) for people living with HIV (PLHIV) in Sub-Saharan Africa over the past 10 years has been remarkable (Bor, Herbst, Newell, & Barnighausen, 2013), and Malawi in particular has substantially reduced HIV-attributable adult mortality(Chihana et al., 2012; Jahn et al., 2008). In the rural north of Malawi, where resource constraints are particularly apparent, the all-cause mortality rate among 15–59-year-olds fell by 32% once ART was introduced, compared to the pre-ART period (Floyd et al., 2010). This dramatic decline is the result of a successful public sector HIV treatment program. Malawi was one of the first sub-Saharan countries to adopt a “public health approach” to HIV scale-up, as promoted by the World Health Organization to encourage rapid ART initiation for PLHIV (Gilks et al., 2006). Despite recent improvements in ART retention, substantial attrition across the HIV diagnosis-to-treatment “cascade” of services continues to result in excess deaths among PLHIV before, and after initiating ART (Jahn et al., 2008; Koole et al., 2014; McGrath et al., 2010; Parrott et al., 2011; Wringe et al., 2012; Zachariah et al., 2006).

The network for Analyzing Longitudinal Population-based data on HIV in Africa (ALPHA) is investigating mortality among PLHIV during the ART era, using longitudinal data from 10 health and demographic surveillance sites (HDSS) in six high prevalence countries: Kenya, Malawi (Karonga), South Africa, Tanzania, Uganda and Zimbabwe. As part of this research program, a study was conducted to understand policy and programmatic factors that facilitate or inhibit PLHIV from accessing and staying on treatment; aiming to investigate whether policy implementation differences could explain variations in the distributions of deaths among PLHIV across the diagnosis-to-treatment cascade.

Earlier work has shown that Malawi has more policies to promote rapid progression of PHLIV through the cascade than other countries (Church et al., 2015). In this paper, we explore national HIV policy and its implementation within the Karonga HDSS in northern Malawi by contrasting policy review findings with health facility survey data to provide rich contextual analysis of HIV policy implementation in this setting.

## Methods

### Study setting

By 2012, there were approximately 950,000 adults aged >15 living with HIV in Malawi (UNAIDS, 2012), corresponding to an adult prevalence of 10.8% (National Statistics Office (NSO), 2011). There were 724 static and 188 outreach testing sites, and 707 static ART sites in mid-2014 (Ministry of Health Malawi, 2014a). By September 2014, an estimated 521,319 adult patients were alive and on ART, and 78% of adults initiating ART were retained in care one year later (Ministry of Health Malawi, 2014a).

The HDSS was established in 2002 in Karonga District, and has conducted HIV serosurveys since 2005. The site is located in a rural area of 135 km^2^, with a population of approximately 37,500. HIV prevalence in the HDSS was estimated at 7% of men and 9% of women in 2012 (Floyd et al., 2012). Free ART became available in Karonga district in 2005, and within the HDSS in 2006.

### Policy review

**Figure 1.**
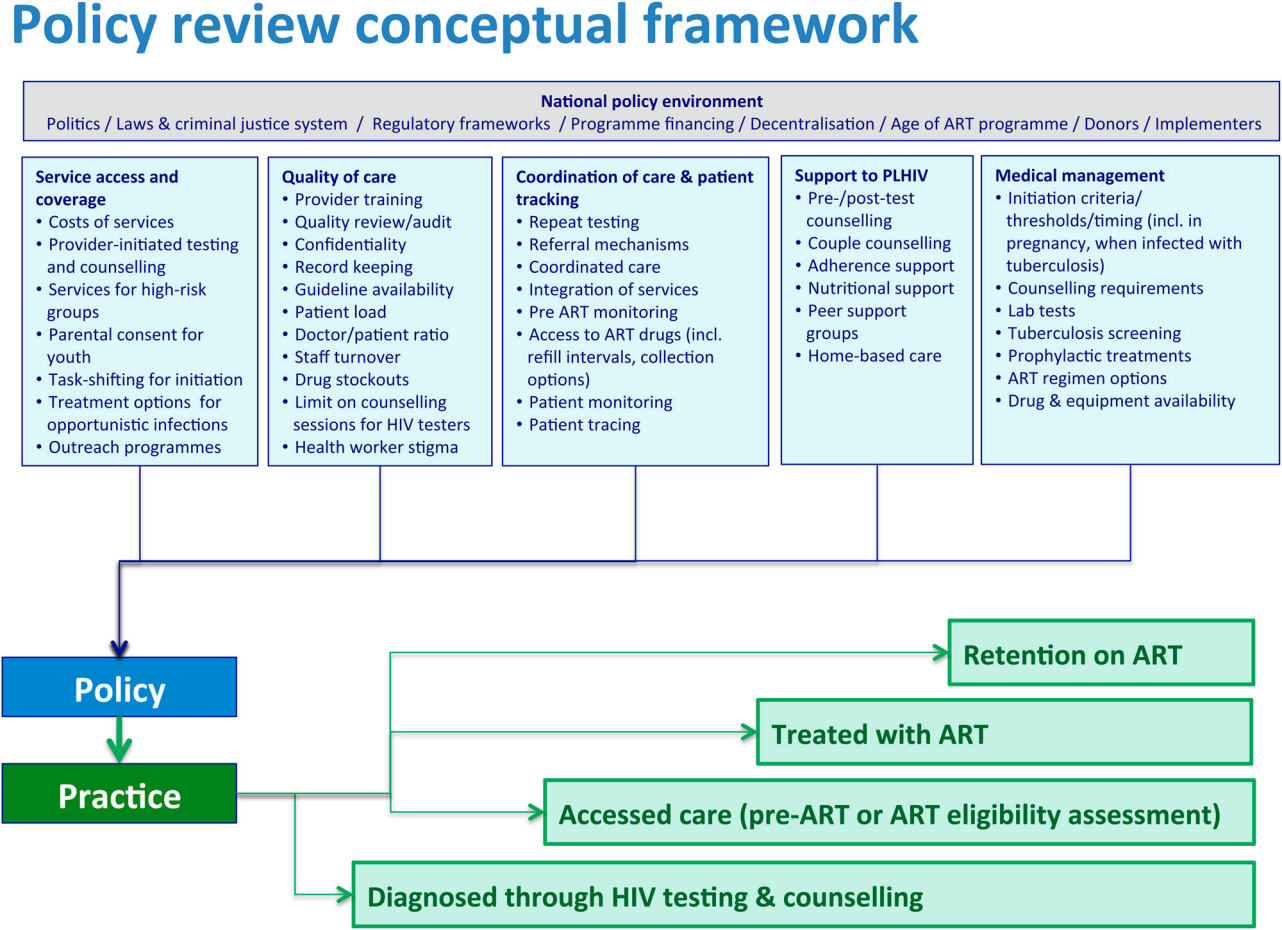
Conceptual framework of policy and service factors influencing adult mortality across the diagnosis-to-treatment cascade. Reproduced from Church et al. (2015).

We applied a policy framework developed by Church et al. (2015; Figure 1), devised by reviewing WHO policies/guidelines and the literature, with expert review by a group of HIV researchers and clinicians. The framework includes 54 indicators describing service coverage and access, quality of care, coordination of care and patient tracking, support to PLHIV and medical management.

We reviewed all national Malawian policies and guidelines published between January 2003 and June 2013 on adult HIV testing and counseling (HTC), prevention of mother-to-child transmission services (PMTCT), and HIV care and treatment. Eighteen documents were reviewed (policy statements, clinical guidelines, training manuals and strategies), and policy information was systematically extracted into an Excel tool, noting policy content, source, year of policy, and changes between 2003 and 2013. Policy data were then synthesized into a series of indicators categorized according to whether they were: (i) explicit; (ii) implicit or with caveats or exceptions; (iii) unclear or conflicted with other policies; or (iv) no policy in this area. Further details on this process are provided elsewhere (Church et al., 2015).

### KI interviews

Three semi-structured interviews with key informants (KI) from the National AIDS Commission, the Ministry of Health HIV/AIDS unit, and a researcher/clinician working on HIV/AIDS at an NGO were conducted to fill in gaps in the document review. KI also provided their views on policy implementation, and these insights helped to shed light on the reality of service delivery. Interviews were audio-recorded, and notes were taken.

### Facility survey

**Figure 2.**
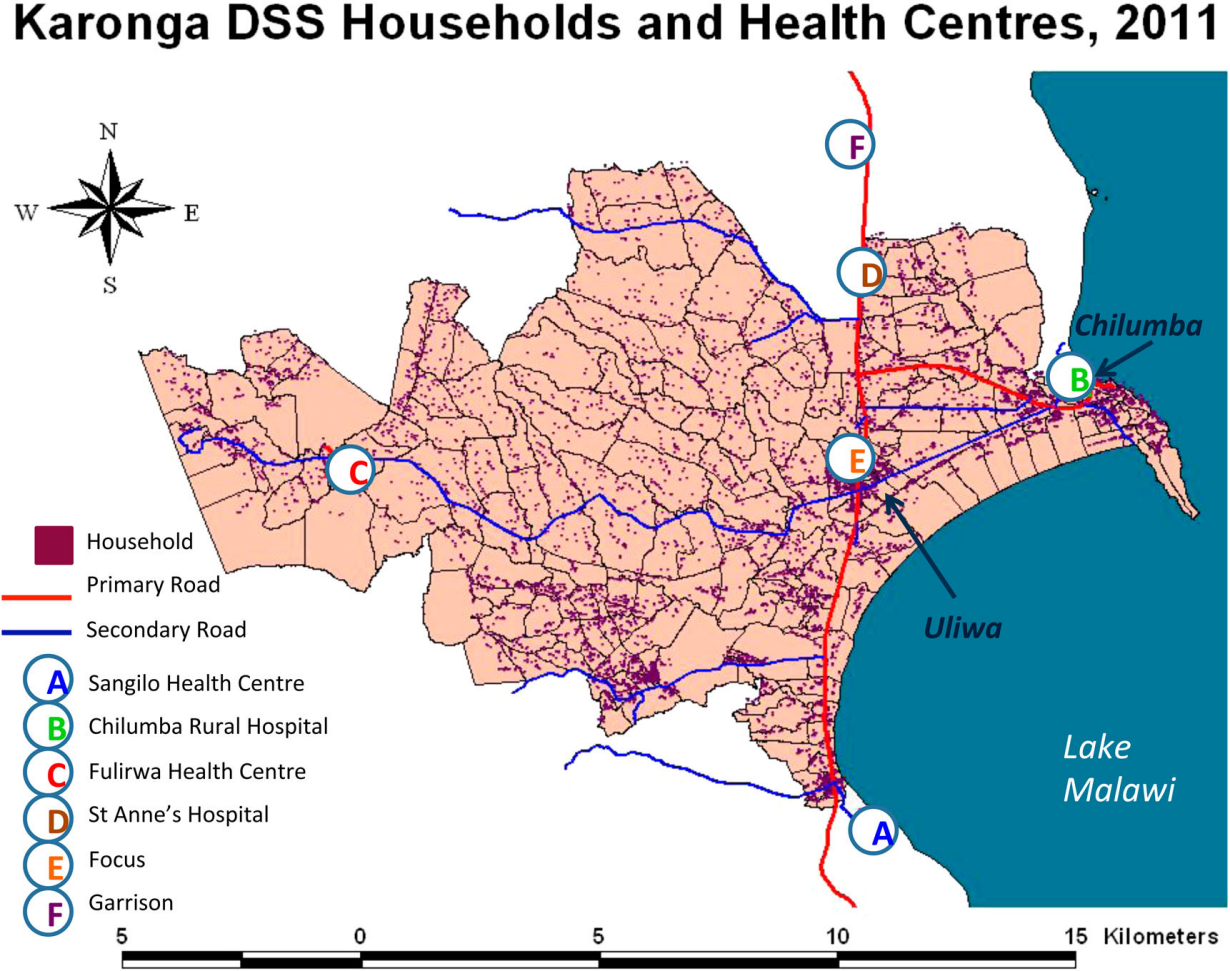
Karonga HDSS and location of health facilities. Copyright Malawi Epidemiology and Intervention Research Unit. Permission to reproduce has been given.

A structured questionnaire was designed to investigate implementation of policy in adult HTC, PMTCT, and HIV care and treatment. The survey was conducted in December 2013 in all six facilities with catchment areas within the Karonga HDSS that provided HIV treatment services (*n* = 5), or community support and HTC services (*n* = 1) (Figure 2). Of the five facilities providing HIV treatment, one was a rural hospital, and four were rural health centers. A nurse interviewer conducted the group interviews in English with the Officer-in-charge and other healthcare providers involved with HIV services at each health facility. Participants gave informed consent, and interviews took around 90 minutes.

Ethical approval for the facility survey was granted by the Malawi National Health Sciences Research Committee. The facility survey data were analyzed descriptively using Stata 12 according to the same 54 indicators that were assessed for the policy review.

## Results

### The national policy response to the HIV epidemic in Malawi

There have been two points of major change in HIV care and treatment in Malawi. The public sector rollout of the ART program in 2004 was groundbreaking, bringing the potential for wide-scale delivery, and the landscape of HIV treatment changed completely. Prior to this, there was ad hoc small-scale private treatment confined to major cities and mostly delivered by non-governmental organizations to a few patients (NAC Ministry of Health Malawi, 2005). The “public health approach” meant patients initiating ART did not require CD4 counts, virology or other laboratory tests, with eligibility assessment based on clinical screening only; those assessed as being in WHO Clinical Stage 3 or 4 were eligible to receive ART.

A second major change in the provision of HIV services occurred in 2011 when the 1st edition of the “Malawi Guidelines for Clinical Management of HIV in Children and Adults” was compiled (Ministry of Health Malawi, 2011). This focused on improving access to PMTCT and ART, including the introduction of the “Option B+” policy in 2011, which recommended that all HIV-positive pregnant or breastfeeding women initiate lifelong ART following diagnosis. Malawi was the first country in sub-Saharan Africa to adopt this policy, even before WHO recommended it in 2012 (World Health Organization, 2012).

The 2011 guidelines also brought in CD4 testing, and indicated for the first time that ART should be initiated for patients with WHO clinical stages 1 and 2, if they also had a CD4 count below 350 cells/mm^3^. Stavudine was phased out and policy recommended tenofovir/lamivudine/efavirenz for new patients, starting with TB patients and pregnant women, and those with lipodystrophy (Ministry of Health Malawi, 2011).

### HIV policy implementation

There were 2–6 participants per interview group in the facility survey. Respondents included: clinical officers, nurse midwives, medical assistants and HTC counselors. Tables 1–3 present information on the indicators, and describe how policies are being implemented in the six facilities, with regard to HTC, HIV care and treatment, and retention in care.

**Table 1.** Access to HIV testing and counseling.

Policy indicator	Year of WHO guideline	Malawi policy	Malawi practice: (i) Karonga HDSS facilities implementation, *N* = 6 offering HIV testing (number of facilities in parentheses) and (ii) insights from KIs
**Service coverage and access factors**			
Free testing at public facilities	2003 implied	HTC, as a service of the Essential Health Package, should be provided free of charge in public-sector facilities and in stand-alone sites (Ministry of Health Malawi, 2006b). CHAM/private facilities liaise with DHO to get testing kits and they do not charge a service fee, although they might charge a consultation fee. If the DHO runs out and the private facility has to buy them elsewhere, there would be a charge to the patient.^a^	**Complies:** In all 6 facilities, services reported as free for patients, including clinic registration fee, HIV test fee, equipment fees.
Provider-initiated testing and counseling (PITC) is standard for all clients including at ANC	2004	Health providers are asked to ascertain HIV status for all patients attending health services (PITC). Patients in ANC are especially encouraged for HTC, due to the Option B+ policy (Ministry of Health Malawi, 2011). The PITC concept was there at the beginning and started with ANC mothers.^a^	**Partial:** All 5 facilities offering ANC, offer PITC. 4 out of 5 facilities offer PITC to OPD, TB, and STI/FP clients. However, 4 facilities noted it was “opt-in” rather than “opt-out” testing.
Testing targeted at high-risk groups (e.g., sex workers, men who have sex with men, injecting drug users)	2004	No specific policy on groups such as MSM, IDU, sex workers. The lack of policy on targeting MSM/sex workers contrasts with a long section on targeting testing to the deaf, dumb and visually impaired (Ministry of Health Malawi, 2006b)	**Unclear:** Testing services are “offered” (note, not “targeted”) to: sex workers (5), MSM (0), drug users (3), prisoners (1), truck drivers (3), factory/estate workers (1). Some moonlight testing takes place, where mobile clinics target sex workers/truck drivers in hot spots.^a^
Parental consent not required for youth testing (<18)	2007	When any person aged 13+ is requesting HTC, they should be considered mature enough to give full and informed consent. Youth aged between 9 and 12 years and are sexually active should be regarded as mature minors and considered eligible to give consent for HTC. The HTC counselor should make an assessment of their readiness for HTC services. HTC for youth below 13 years of age should be done with the knowledge of their parents/guardians, unless this is not possible and the testing is for provision of treatment and care services.	**Complies:** Testing services are offered to schoolchildren in six facilities.
**Quality of care factors**			
Anonymous HIV testing		No names recorded in HTC register. However, ART, TB, and ANC registers have names and HIV test results. For in-patients, the test result is documented in in-patient notes. All patient cards and clinic registers are the property of the Ministry of Health (MoH) and may only be kept at the respective facility or at the National Archives. They must be kept in a locked room and only accessed by clinic staff responsible for providing the respective service and by the national supervision team (Ministry of Health Malawi, 2011).	**Not implemented:** All facilities report they record patient HIV test data in registers/logbooks. One facility also records on patient-retained cards. The data collected are: sex, age/DOB, residence, name and test result.
Counselor counsels 15 clients/day max		Not specified	**Unclear:** 3 facilities reported there is no maximum. 2 facilities reported max 8. 1 facility reported max 10.
Periodic refresher training for counselors required	2001 onwards. No frequency	No clear guidelines. No new counselors are being recruited until refreshers have been done for all HTC counselors. This is because there were concerns over quality of service, so refresher trainings were prioritized.^a^	**Exceeds:** 26 medical staff (doctors, clinical officers, nurses, trained counselors) provided HTC services. Out of these, 23 received formal training in HIV testing within the past 2 years.
Periodic quality control checks at testing sites required	2005. No frequency	Activities to maintain and strengthen quality of HTC services include an internal and external quality assurance for rapid HIV testing by laboratory technologist and proper documentation and dissemination of QA procedures and protocols to testing service providers, mentorship and supervision of services, client satisfaction surveys, mystery client surveys, awards/prizes/rewards, centers of HTC excellence etc. (Ministry of Health Malawi, 2006b)	**Complies:** Quality of care reviews/audits of HIV testing are conducted once every 3 months (3 facilities), once a month (2), and once a year (1).
**Coordination of care and patient tracking factors**			
Negatives retest every 6–12 months	2007	Re-testing every 6–12 months is beneficial for individuals at higher risk of HIV infections, e.g., persons with a history of STI, sex workers and their clients, and sexual partners of PLHIV (Ministry of Health Malawi, 2009). The policy might change such that low-risk people (repeat HIV-) can leave a longer space between each test (economizing use of test resources).^a^	**Unclear:** People who test HIV negative are advised to test again in 3 months (3 facilities), or test when they feel worried (3 facilities). It is unclear if the window period was misinterpreted as repeat testing, but if so, this is more frequent than policy. Testing when worried is ambiguous.
Repeat testing in 3rd trimester of pregnancy (if previously test negative)	2010	An HIV test should be provided on 1st ANC visit.^a^ It is also stated that HIV- women’s status should be considered unknown after 3 months window period (Ministry of Health Malawi, 2010). Therefore, if the woman attended ANC early in pregnancy, she would have repeat testing in the 3rd trimester.	**Partial:** All 5 facilities reported women get tested at first ANC visit for this pregnancy. Only 2 out of 5 facilities said the woman gets tested at their 3rd trimester visit.
**Support to PLHIV factors**			
Individual as well as group pre-test counseling recommended	2003	Pre-test counseling is a requirement, either individually or group (Ministry of Health Malawi, 2006b, 2010). If done in a group there should be between 5 and 15 people in a group, and attempts should be made to ensure that the group is homogenous in relation to age and sex, and is culturally appropriate (Ministry of Health Malawi, 2009).	**Complies:** Pre-test counseling “always” given in 5 out of 6 facilities, and “usually” given in the 6th facility. All 6 sites do individual pre-test counseling. 5 out of 6 sites do group pre-test counseling.
Couple counseling encouraged	2004	Patients encouraged to attend HTC with their marital/sexual partner (Ministry of Health Malawi, 2006b, 2011). It was also planned that there should be at least 1 counselor at each testing site with specialist couple counseling skills (Ministry of Health Malawi, 2006b).	**Complies:** VCT for couples is offered in all 6 facilities.

CHAM=Christian Health Association of Malawi, DHO=District Health Office, MSM=Men who have sex with men, IDU=Injecting Drug User, TB=tuberculosis, OPD=Out patient department, STI=Sexually transmitted infection, DOB=Date of birth.

^a^Source: Key informant interview.

**Table 2.** Access to HIV care and treatment.

Policy indicator	Year of WHO guideline	Malawi policy	Malawi practice (i) Karonga HDSS facilities implementation, *N* = 5 offering PMTCT and ART (number of facilities in parentheses) and (ii) insights from KIs.
**Service coverage and access factors**			
Free PMTCT at public facilities	“Universal access”	PMTCT free at government facilities. Service-level agreements between MoH and CHAM makes it free of charge at CHAM.	**Complies:** All 5 facilities do not charge anything for PMTCT services (clinic registration, service fee, drugs, equipment). In practice, patients may have to pay a prescription fee at CHAM facilities, but they are not supposed to.^a^
Free ART at public facilities	“Universal access”	ART to be provided free at point of delivery in public sector and CHAM to eligible persons (Ministry of Health Malawi, 2006a, 2006c). In private sector, ARV drugs will be subsidized, with patients paying roughly equivalent of US $4 (NAC Ministry of Health Malawi, 2005). Delivery of ART through private sector is to take load from public sector, thereby allowing it to concentrate on the free system.	**Complies:** All 5 facilities do not charge for HIV care and treatment services, including clinic registration, HIV care and treatment service fee, fees for drugs, equipment fees, laboratory tests.
PMTCT available at all ANC facilities	2010	In ANC, there should be a range of PMTCT activities including HTC for mother, siblings and partners, dispensing of ARVs for mother and baby, CD4 count and staging (Ministry of Health Malawi, 2010),	**Complies:** All 5 facilities providing ANC also provide PMTCT services.
Clinical officers, medical assistants and/or nurses initiate ART		All certified clinical PMTCT/ART providers are authorized to initiate, prescribe and dispense ART (doctors, clinical officers, medical assistants, registered nurses, nurse/midwife technicians). They need to have (a) attended a pre-service ART training module and passed the final exam, or (b) attended an ART training course recognized by MoH, Medical Council of Malawi and Nursing Council of Malawi and passed an exam (Ministry of Health Malawi, 2008).	**Complies:** All 5 facilities have nurses that can initiate. 3 facilities have clinical officers that can initiate. 1 facility has a medical assistant that can initiate.
All sites providing ART also initiate ART		All sites that provide ART also initiate ART. Facilities only provided with ARV drugs if formally assessed by MoH as ready to deliver ART. Readiness criteria include (a) plans for recruitment and follow-up of patients, (b) functioning CT services (c) dedicated room for ART delivery, equipped and has monitoring tools and copies of guidelines (d) trained staff and (e) secure storage for ARV drugs.	**Complies:** All 5 facilities providing ART also initiate ART.
**Coordination of care and patient tracking factors**			
HIV-positive clients followed-up to ensure registration at treatment site	2004	No clear follow-up policy. Counselors should have a directory of HIV-related prevention, treatment, care and support services available for clients in catchment area. Patients’ HIV test results and names will be documented for such referrals (Ministry of Health Malawi, 2009).	**Exceeds:** Referral to HIV care and treatment services is documented in (a) patient notes (5 facilities), (b) recorded on a referral form (1), (c) referral letter with patient (3), register/logbook (2). Three facilities check if the HIV-positive patients register in HIV care/treatment services. Internal referrals (pre-ART, monitoring, ART initiation, ART resupply) are documented by register/logbook (3), referral letter (2), facility-held patient cards/notes (2), patient-retained cards (3).
Clear guidance on when HIV+ pregnant women be referred to ART clinic	2006	At the time of change to Option B+ (mid-2011), it was left to sites to decide whether to refer women to ART or to continue treatment in ANC, that is, no clear guidelines. In mid-2012, the MoH took stock of retention and found that the model of referral to ART did not work. New guidance stated that ART initiation and follow-up during pregnancy should be in ANC. After delivery, referral to ART still varies.	**Unclear:** Referral to ART clinics from ANC occurs in 3 facilities. Referral after delivery, and referral on the same day, occurs in 1 facility each, respectively. 4 facilities report that a health worker accompanies the woman to ART provider/unit. One facility reports sending the woman to ART provider/unit by herself. Many keep providing ART to women post-partum up to 6 months before referral to ART.^a^
6 monthly CD4 testing in pre-ART with CD4<500		Repeat CD4 counts for patients over 5 years in pre-ART follow-up every 6 months. Move to 3-monthly CD4 counts if last count was less than 500. Stop CD4 monitoring once patient is eligible for ART (Ministry of Health Malawi, 2011).	No data in facility survey.
**Medical management factors**			
WHO “Option B+” is standard (2012) (all HIV+ pregnant women initiate life-long ART)	2012	Option B+ since July 2011. TDF + 3TC (or FTC) + EFV (Ministry of Health Malawi, 2011).	**Partial:** 4 facilities reported that all HIV+ pregnant women initiate. Regimen is TDF/3TC/EFV, for life. 3 facilities provide treatment in same building as ANC but different room. 2 facilities provide in same facility as ANC but different building. All facilities provide treatment on same day as ANC services.
All patients with TB eligible for ART initiation	2009	Since the beginning of the program, Malawi made TB a stage 3 condition. It was not a WHO stage 3 condition, so Malawi went beyond WHO guidelines, and now WHO have followed.^a^	See cell below.
Co-infected TB/HIV should initiate ART on same day or within 2 weeks of starting TB treatment	2013 (ASAP within 8 weeks)	Initiate ART (regimen 5A) within 14 days of diagnosis of active TB. TBT and ART can be started on the same day if patient is stable. (Ministry of Health Malawi, 2011). There was not clear guidance on timing prior to 2011. It was reasonable to start around the end of intensive phase of TB treatment (around 2 months) in order to avoid drug interactions.	**Partial:** 4 facilities say that TB medication and ART can be initiated together, when patients present with TB.
Initiate ART at WHO stage 3/4; or 1/2 with CD4<=350	2009	Initiate ART when WHO stage 1/2 and CD4 ≤ 350, or, WHO stage 3 or 4, regardless of CD4 count (Ministry of Health Malawi, 2011). Prior to 2011, it was a CD4 count of <250.	**Partial:** All 5 facilities said they’d initiate ART if clinical stage 3 or 4. 4 facilities said they’d initiate ART if CD4 count<350.
Initiate ART within 7 days of ART eligibility		Patients who are clinically stable should start ART no later than 7 days after being found eligible (Ministry of Health Malawi, 2011).	**Unclear:** 3 facilities said 2 separate visits to the clinic are required between ART eligibility and receiving ART drugs. 2 facilities said 1 visit is required. No time periods specified.
Lab tests not required to start ART (e.g., FBC, LFTS/RFTS)	Strongly recommended	Just clinical staging and CD4 testing, are needed (and a confirmatory HIV antibody test to rule out any possibility of mix-up of test results or fraudulent access to ART). FBC/LFTS/RFTS not required.	**Complies:** All 5 facilities reported that LFT, RFT, FBC are not required before a patient can initiate ART at this facility.
Adherence counseling not compulsory before ART initiation	Strongly recommended	All patients must receive (a) individual counseling at ART initiation and (b) group counseling 0–5 days before day of initiation. Option B+ women who start ART on same day are allowed to have counseling on a later day.	**Partial:** Before initiating ART, 4 facilities require at least 2 adherence counseling sessions, and one facility requires at least 1 adherence counseling session.

TDF = tenofovir, FTC = emtricitabine, EFV = efavirenz, FBC = Full blood count, QA = quality assurance.

^a^Source: Key informant interview.

**Table 3.** Factors influencing retention in care.

Policy Indicator	WHO	Malawi policy	Malawi practice: (i) Karonga HDSS facilities implementation, *N* = 5, and (ii) insights from KIs^a^
**Service coverage and access factors**			
ART clinic does not have to include doctor or clinical officer		Minimum staff requirement includes 1 clinician (2006) (Ministry of Health Malawi, 2006a)	**Partial:** All 5 sites had at least one clinical officer or medical assistant
**Quality of care factors**			
Periodic refresher training for ART staff required	2010	1-day ART classroom refresher training course to be taken once a year, followed by 1-day knowledge dissemination and best practice. To be organized at regional/zonal level (2006) (Ministry of Health Malawi, 2006b)	**Not implemented:** Out of a total of 43 staff, 15 had received formal medical/nursing training in HIV care and treatment in the past 2 years.
Periodic quality control checks at ART clinics required	2010	Team of experienced clinicians to go to every ART site every quarter, as supervision. They create a list of action points to be followed up. The form has a trigger to call a mentoring team, in case of problems. A certificate of excellence – motivation system – is awarded to “good” sites.^a^	**Complies:** 4 out of 4 facilities said quality of care reviews / audits of HIV treatment services were conducted every 3 months. However, the nature of “quality of care review” is not known, nor whether it is as comprehensive as policy demands.
**Coordination of care and patient tracking factors**			
Routine 6 monthly CD4 count monitoring on ART	Not a requisite	Routine scheduled CD4 monitoring of patients on ART is not supported by the national program (2011), to prioritize pre-ART follow up (Ministry of Health Malawi, 2011). Previously, it was recommended if capacity was there. But this led to limited capacity being used by a handful of sites with no tangible impact.	**Partial:** Two facilities conduct no CD4 tests once stabilized on ART. Two facilities report conducting them every 6 months. One facility does not do CD4 test routinely, but “done if requested”.
3 monthly drug supplies once stable on ART		Patients initiating 1st line ART reviewed after 2 weeks, then every month for first 6 months. Thereafter, stable and adherent patients can be given up to 3 months. In exceptional cases, up to 12 months of ARVs can be dispensed. Patients starting 2nd line ART must be seen every 4 weeks for first 6 months, thereafter, up to 2-month appointments. (2011) (Ministry of Health Malawi, 2011)	**Partial:** All 5 facilities said patients must return for refills every 2 months max, once they stabilize on ART
Pill counts at every visit		The emphasis was previously on the pill count (Ministry of Health Malawi, 2003). Now, the focus is on a formal active dialog (including an assessment and counseling). Ask “Have you had any problems taking your ARVs? Were there any days when you did not manage to take all your tablets at the right time?” Check “Next appointment date” on patient card to confirm patient is not late. (Ministry of Health Malawi, 2011)	**Not implemented:** All facilities check and count patients’ pillbox. Only two facilities also ask patients about pill-taking. * *
Home visits following signs of poor adherence		No policy regarding home visits. However, conduct follow-up group counseling and individual counseling if any sign of poor adherence. Give practical advice: (a) build ARVs into daily routine, (b) ask family or friends for reminders, (c) set a daily alarm on cell phones and (d) keep a “drug diary” and mark every tablet taken. (Ministry of Health Malawi, 2011)	**Complies:** For patients who present with low drug adherence, 1 facility insists on participation in support groups, and all 5 facilities provide psycho-social counseling.
Home visit or telephone contact for missed visits	2013	Patients late for ART appointment to be actively followed from the clinic (home visit, phone, guardian). Patients asked for consent for active follow-up at time of initiating ART (can withdraw consent any time). Prioritize patients on ART and HCC patients eligible to start ART. (2011) (Ministry of Health Malawi, 2011)	**Partial:** For patients that default, one facility does nothing, 3 facilities try to reach the patient by phone and 4 report to do home visits. For patients that are lost to follow up, 2 facilities do nothing, 2 facilities visit at home (1 missing).
**Medical management factors**			
IPT for all HIV+ patients without active TB	2010	Give IPT to all HIV infected who are not on ART, regardless of clinical stage/CD4 count, who don’t have active TB. Start at enrollment for pre-ART follow-up and continue for as long as patient is in pre-ART follow-up. Stop IPT when ART is started (2011) (Ministry of Health Malawi, 2011).	**Complies:** All facilities offered IPT in pre-ART phase, and all currently had IPT in stock.
TB screening at every pre-ART and ART visit	2010	Yes. Screen all patients at every visit (pre-ART and ART) for signs of active TB using 4 standard questions (cough, fever, night sweats, weight loss/failure to thrive). If 1+ signs, thoroughly investigate further (2011) (Ministry of Health Malawi, 2011)	**Partial:** 3 out of 5 facilities screen for TB for all patients *initiating* ART. For those that don’t they would screen based on clinical symptoms of TB. Patients *on* ART are screened for TB in 4 out of 5 facilities.
WHO 1st line ART as standard	2010	D4T/3TC/NVP (Ministry of Health Malawi, 2011). But plans to switch.	**Exceeds:** All facilities providing TDF+3TC+EFV * *
At least four 1st line regimens choices in national programs	2006	There are 6 different 1st line regimens. 3 are used for initiating ART. All are fixed-dose combinations (only 1 type of tablet). Move patients with significant side effects to an alternative 1st line regimen. (2011) (Ministry of Health Malawi, 2011)	**Partial:** Variable. Number of different regimens in 4 of the facilities: 2, 2, 4, 6. Missing data for 5th.
**Support to PLHIV factors**			
At least one adherence counseling conducted individually		All patients must receive individual counseling at ART initiation. In addition, patients should attend an ART group counseling session between 1–5 days before the day of ART initiation, or on same day as ART initiation. Patients must attend group counseling with named guardian (or treatment supporter). (2011) (Ministry of Health Malawi, 2011).	**Complies:** 4/5 fully adhere to Malawi policy. One facility just does individual counseling only.
All patients on ART referred to peer support groups		No MoH policy. It has not been proactive in pushing this. Left to the site, and their HSAs.	**Exceeds:** No question asked about referrals, but 3 facilities said there were support groups for PLWH in the community: 1 at another facility, 1 ran the support-group at the facility itself
Nutritional supplements for malnourished patients	2006	None. There used to be an HIV unit attempt at providing adult food interventions (plumpy-nut), but it was found to be expensive and it didn’t show a measureable impact. It was decided to treat the underlying condition (HIV) and this should address the malnutrition. So adult food supplementation was taken out of clinical guidelines.^a^	**Unclear:** 3 facilities provide food-packages/nutritional support to patients. 2 facilities say that food-packages/nutritional support is available at another facility. But these were child nutrition programs.
All patients on ART referred to home-based care		Very little on home-based care, and not handled by HIV department. If anything, it is self-organized by individual sites, or NGOs.^a^	**Exceeds:** All 5 facilities claim home-based care is provided either by them (2), another facility in the area (2), or at the community level (1)

^a^Source: Key informant interview.

### Practice complies with policy guidelines, fully or partially

For some indicators, practice complied with national policies. For example, in all surveyed facilities, free HTC (Table 1), PMTCT and ART (Table 2) services were reported. Individual, group and couple pre-test counseling is conducted in most facilities, which aligns with policy (Table 1). Provision of free HIV services was not restricted to government facilities: service-level agreements with faith-based facilities extended coverage of free HIV care, in line with policy (Tables 1 and 2).

PMTCT is available at all five antenatal care (ANC) facilities (Table 2). Quality of care reviews are routinely conducted (Tables 1 and 3), although it was not clear whether these were as comprehensive as policy indicated (Table 3). No laboratory tests were required for PLHIV to initiate ART, in line with policy.

Policies to promote rapid initiation were implemented including task sharing to nurses and medical assistants, and rollout of ART initiation to all facilities (Table 2), thus making treatment rapidly accessible for many more people. All facilities offered isoniazid preventive therapy (IPT) in the pre-ART phase, and all had Isoniazid in stock at the time of survey (Table 3). All facilities provided some form of counseling following signs of poor adherence (Table 3) (pill counts and active dialogue are supposed to be employed to identify signs of poor adherence).

In some instances, policy was only partially implemented. For example, while provider-initiated testing and counseling (PITC) was national policy, and all facilities with ANC offered it to pregnant women, only four facilities routinely offered HTC to other groups (e.g., out-patients, TB and family planning clients), and four facilities only offered “opt-in” testing (Table 1). ART initiation through Option B+ policy was implemented in 4/5 facilities offering ART (Table 2), and ART patients were given two-monthly drug supplies once stable, not three as indicated by the policy (Table 3). Variations across facilities were observed in procedures following missed visits, treatment defaults and TB screening, and the number of first-line regimens offered also varied (Table 3).

### Policy not implemented, or unclear

Indicators of practice not adhering to policy included: infringement of anonymous HIV testing by recording names in registers (Table 1); referral of HIV-positive pregnant women from ANC to the ART clinic during pregnancy in spite of clear guidance since 2012 that women should initiate and be maintained on treatment within ANC until after delivery (Table 2); low provision of refresher training for HIV care providers (Table 3); limited diligence in investigating adherence (Table 3). While all facilities did report pill counts to monitor adherence, the failure to ask patients specifically about pill-taking suggests that the move towards an “active dialogue” as outlined in the policy was absent.

### Practice exceeds policy guidelines

Although Malawi had policy gaps in relation to ART registration, adherence and quality of care, the facility surveys demonstrated that there were often alternative practices being implemented, which focused on adherence and quality of care. For example, although there was no clear policy guidance on referral procedures to link diagnosed clients with ART sites, facilities reported a range of strategies to track patients’ post-diagnosis and ensure registration in HIV care services. Five facilities reported documentation of referral in patient notes, three provided referral letters to patients, two completed registers/logbooks, and one used a referral form. Furthermore, three facilities reported checking HIV care registers to ensure that registration occurred. A healthcare worker accompanied clients to the ART unit in two of the facilities to ensure registration (Table 2).

Similarly, while there were policy gaps regarding home-based care and peer support, other policies and practices may have compensated for this. For example, Malawi policy indicates that patients must have follow-up counseling following signs of poor adherence, and be given clear practical advice (Table 3). Policy documents also stated that patients should be asked to consent to active follow-up at the time of initiating ART. Facilities themselves also took the initiative in this area; three reported support groups for PLHIV in the community, and all five reported home-based care was provided either by them, another facility, or at the community level, although these programs were small scale and dependent on local circumstances.

While there was no specific national policy on targeted testing of high-risk groups, most facilities offered testing to some groups, notably sex workers. A key informant also noted that mobile clinics targeted sex workers and truck drivers in hot spots. Clinics had also preceded national policy in the delivery of first-line ART regimens. At the time of the survey, national policy stipulated D4 T/3TC/NVP (Stavudne/Lamivudine/Nevirapine) provision, yet all five facilities with ART were already delivering a tenofovir-based regimen (made available due to the Option B+ policy for pregnant or breastfeeding women), which subsequently became policy for all adults.

## Discussion

Our policy review demonstrates that the Malawian government has taken a groundbreaking public health approach to developing national HIV policies in order to encourage as many PLHIV as possible to access and adhere to treatment. Furthermore, these policies covering testing, PMTCT and retention in HIV care have generally been successfully implemented in facilities in this poor, rural and remote setting. However, despite a general picture of successful implementation, we documented several areas of disconnect between policy and practice that are likely to contribute to attrition along the diagnosis-to-treatment cascade, and attention to these could improve retention in care.

For example, the observation that healthcare providers continued to rely on the pill count rather than engage in active dialogue with the patient is concerning, although likely explained by the additional workload and lack of complex provider skills. Furthermore, as the MoH had identified that immediate referral from ANC to ART for pregnant women was not effective, the fact that several facilities continued to make this referral as soon as pregnant women were found to be positive could explain why some drop out of care. A separate analysis found that shortly after Option B+ implementation, almost half of HIV-positive pregnant women in the HDSS and not already on ART when they became pregnant had not initiated lifelong ART by the time of delivery (Price, 2014). Similarly, there was lower retention in care among women initiated on ART because of Option B+, compared to women of childbearing age initiated on ART because of clinical or CD4 cell count criteria (Koole et al., 2014).

In some instances, failure to comply with national policy may have contributed to improved rates of linkage to care. For example, while an anonymous HIV testing policy may have been designed to reduce stigma and fear of disclosure, recording names in HIV testing registers may promote patient tracking, identification and entry into care. An unexpected finding in this study was the discovery that several health facilities were implementing activities that went beyond national policies. For example, we documented good referral services from HTC and ANC services to the ART clinic in the facility survey, despite a lack of policies on linking positive individuals from testing services to HIV care and treatment. It has been shown that progression from HTC to ART is comparatively fast (Reniers et al., 2015), and this is likely due to these enhanced procedures, although it may also reflect the exposure of this population to the HIV serological surveys which include opportunities for HIV testing and referrals.

Our analysis has indicated that many of Malawi’s progressive policies are implemented, for example, most women initiate ART in line with the Option B+ policy, although their impacts on retention and mortality remain uncertain. For example, Option B+ resulted in a 49% increase in ART coverage among HIV-infected pregnant women in 18 months (Tippett Barr, 2013), but treatment default rates among pregnant or breastfeeding women are higher than among non-pregnant women, with Option B+ patients five times more likely to never return after their initial clinic visit than women starting ART with advanced disease progression (Tenthani et al., 2014). These findings suggest that further research is needed to investigate the impact of Option B+ policy implementation on adherence, drug resistance rates, and mortality.

Since the policy review was conducted, a second edition of the Malawi Guidelines for the Clinical Management of HIV in Children and Adults was published in April 2014. These took a new direction with emphasis on better monitoring using viral load testing, to improve identification of treatment failure and ensure timely initiation of second-line treatments and further improved quality of care. These guidelines also indicated that ART should be initiated in adults with CD4 counts <500 (Ministry of Health Malawi, 2014b) even in the absence of AIDS-defining illnesses, moving closer to a test-and-treat approach.

The approach taken in this work, drawing on qualitative policy data, quantitative facility survey data, and interviews with key informants, has helped to inform review of HIV policy and its implementation. This model may be useful in other settings. Similar analyses are being conducted in other ALPHA sites as well as a review across countries (Church et al., 2015). Findings have been shared with the Malawi Ministry of Health HIV Unit (through author SM) and Karonga District Health Office (through author CS).

There are certain limitations to this analysis that must be considered. Firstly, the facility surveys only covered six facilities in a small area in northeast Malawi, and therefore our findings are not representative of the whole country. Certain findings from the facility survey were, however, supported by our KI interviews, suggesting that the findings from the facility survey are not unique to the study area. In this respect, KIs bridged the information between policy (from the review) and practice (from facilities) as they had insights to share on both.

Secondly, healthcare providers may have thought that they were being audited and presented their work and facility in an optimal light. Despite an explicit introduction by the interviewers, respondents may have thought that they should answer what is *supposed* to happen according to their own training, rather than what happens in reality. We tried to limit this by providing detailed information on the purpose of the study, and stressing the anonymity of the results before carrying out the interviews.

Finally, no data were collected from patient interviews. Patients’ experiences of implementation are likely to vary and differ from provider reports. Our KIs suggested, for example, that patients may be charged prescription fees, which was not reported in our surveys. Qualitative interviews with PLHIV across the cascade are being conducted to better understand their perspectives on HIV service delivery and how different policies influence care-seeking behavior. Future analyses should also quantify which policies and practices are most effective in improving retention in care and reducing mortality, by incorporating mortality data among HIV-infected persons in this setting.

This analysis demonstrates where national policies are being implemented in health facilities in northern Malawi; and identifies policy implementation gaps as well as where practices exceed national recommendations. Addressing areas of shortfall, while reinforcing areas of excellence, could improve retention across the diagnosis-to-treatment cascade and reduce mortality among PLHIV.

## Addendum

Since the submission of this article, a viral load monitoring plan has been launched in Malawi, together with a commitment to move towards the WHO recommendation for immediate treatment.
